# ‘Every day was a learning curve’: implementing COVID-19 triage protocols in UK ambulance services—a qualitative study of staff experiences

**DOI:** 10.1136/emermed-2024-214495

**Published:** 2025-09-17

**Authors:** Alison Porter, Fiona Bell, Mike Brady, Shona Brown, Andrew Carson-Stevens, Timothy Driscoll, Bridie Angela Evans, Theresa Foster, John Gallanders, Imogen Gunson, Robert Harris-Mayes, Mark Kingston, Ronan Lyons, Elisha Miller, Andy Rosser, Aloysius Niroshan Siriwardena, Robert Spaight, Victoria Williams, Helen Snooks

**Affiliations:** 1 Swansea University Medical School, Swansea University, Swansea, Wales, UK; 2 Yorkshire Ambulance Service NHS Trust, Wakefield, UK; 3 Welsh Ambulance Services NHS Trust, Cwmbran, UK; 4 East of England Ambulance Service NHS Trust, Melbourn, Cambridgeshire, UK; 5 Division of Population Medicine, School of Medicine, Cardiff University, Cardiff, UK; 6 SUPER Group PPI, Swansea, UK; 7 West Midlands Ambulance Service NHS Trust, Brierley Hill, West Midlands, UK; 8 Lincoln School of Health and Social Care, University of Lincoln, Lincoln, England, UK; 9 Clnical Audit and Research Unit, East Midlands Ambulance Service NHS Trust, Nottingham, UK; 10 University of Bristol, Bristol, UK

**Keywords:** COVID-19, triage, pre-hospital, qualitative research, operations research

## Abstract

**Background:**

TRIM (What TRIage model is safest and most effective for the Management of 999 callers with suspected COVID-19? A linked outcome study) was an evaluation of models used to triage and manage emergency ambulance service care for patients with suspected COVID-19. In an embedded qualitative component, we aimed to understand experiences and concerns of clinical and managerial staff about processes for responding to patients with suspected COVID-19, in the call centre and on scene.

**Methods:**

Research paramedics in four study sites across England interviewed purposively selected stakeholders from ambulance services (call handlers, clinical advisors in call centres, clinicians providing emergency response, managers) and emergency department clinical staff. Interviews (n=25) were conducted remotely, recorded and transcribed. Thematic analysis was conducted by a group of researchers and PPI (patient and public involvement) partners working together.

**Results:**

We present four themes, developed from the data. Services made efforts to target their response to those most in need, while trying to minimise infection risk; they reduced face-to-face contact where possible, dealing with more calls remotely. Adjustments by other providers in the wider healthcare system affected the flow of patients to and from ambulance services. There was substantial work and heavy cognitive load for staff at all levels in updating knowledge and repeatedly implementing changes. Staff working in the range of roles in ambulance services also carried a heavy emotional load.

**Conclusions:**

Services made flexible changes to triage processes using the best level of understanding available at the time, in a healthcare setting which always operates in high levels of uncertainty. Implementing triage protocols in response to the COVID-19 pandemic was a complex and fluid process which had to be actively managed by a range of front-line staff, dealing with external pressures and a heavy emotional load. Increased understanding of the way in which services and staff had to adapt, and the cognitive and emotional burden this entailed, may help in planning for future pandemics.

**WHAT IS ALREADY KNOWN ON THIS TOPIC**

- Like all health services, emergency ambulance services had to rapidly adapt their response to meet the demands of the COVID-19 pandemic.

**WHAT THIS STUDY ADDS**

- We provide qualitative insights into the burden on staff of providing rapidly changing responses in triage and decision-making practice in emergency ambulance services, against a backdrop of the heavy emotional load of working through the pandemic.

**HOW THIS STUDY MIGHT AFFECT RESEARCH, PRACTICE OR POLICY**

- Future pandemic responses should acknowledge the burden on staff and the risk of moral distress or injury.

## Introduction

 The COVID-19 pandemic presented emergency ambulance services worldwide with an unpredictable challenge in meeting service demand.^1^,^5^ Ambulance services in the UK introduced changes to their usual processes for triaging and managing emergency responses, against the backdrop of evolving national guidelines and emerging evidence about the risks and impact of COVID-19.^6^

The qualitative work reported here was part of TRIM (What TRIage model is safest and most effective for the Management of 999 callers with suspected COVID-19? A linked outcome study), a mixed-methods evaluation of UK emergency ambulance service responses for patients with suspected COVID-19 during the first wave of the pandemic in 2020. Although a recent scoping review^7^ of Emergency Medical Services (EMS) interventions and experiences during pandemics identified 90 studies, of which 7 were from the UK, the majority of these took a quantitative approach to assessing the impact of changes in practice. In this paper, we take a qualitative approach to examining how ambulance service staff enacted these adaptive responses to the pandemic, and how they, and clinical colleagues in emergency departments, experienced these changes to their working practice.

## Methods

### Setting

The qualitative component of TRIM was conducted in four diverse UK sites, each consisting of one regional emergency ambulance service together with the associated healthcare economy.

Services entered the pandemic with established procedures, based on one of two call categorisation and prioritisation systems: Medical Priority Dispatch System (MPDS)^8^ and NHS Pathways.^9^ These systems guide call handlers in Emergency Operations Centres (EOCs) through decision pathways, using standard questions and prompts, leading to the patient being assigned a priority category for the service response. An EOC clinical advisor (a paramedic, nurse or doctor) might advise on certain calls, or review ones waiting as low priority, to suggest what to do or to give advice directly to the patient. Ambulance crews attending patients make decisions, with the patient and informed by national guidelines, about whether to convey the patient to hospital, which hospital, whether to pre-alert and what care to give directly.^10^

Changes in ambulance service practice took place in terms of triage and decision-making relating to prioritisation, dispatch and conveyance, including protocol-driven primary triage in the call centre; remote secondary triage by clinicians of a portion of calls; and conveyance decisions by clinicians at scene. A pandemic protocol, known as Card 36, for coding a patient as ‘suspected COVID-19’ was brought into use in AMPDS in early April 2020. Within NHS Pathways, there was no specific protocol for responding to patients with suspected COVID-19, although some changes to triage practice were introduced. In response to prevailing levels of infection, the thresholds for different types of response were adjusted, for example, to increase the proportion of calls resolved with telephone advice. Analysis of routine data in TRIM showed that emergency calls for suspected COVID were more likely to result in ambulance dispatch, but less likely to lead to conveyance of the patient to hospital, compared with non-COVID calls.^11^

### Data collection and analysis

The study took place in four sites across England, each consisting of one emergency ambulance service together with one hospital emergency department (ED) to which that service conveyed patients. One research paramedic was recruited in each study site; all four were female, with experience of qualitative research. We prepared two interview schedules, one for ambulance service staff and one for ED staff. Interviews (n=25) took place in March–August 2021. Under the guidance of the study manager and in line with selection criteria agreed by the study team, research paramedics recruited participants by emailing invitations, along with a participant information sheet and consent form. Each research paramedic was asked to recruit at least six participants in a specified range of roles, in order to provide diversity of experiences within sites but consistency across sites. The approach was purposive and pragmatic, making use of existing networks, and only the research paramedics had access to identifiable information on the participants. Interviews were conducted remotely using MS Teams, and recorded and transcribed in full, and anonymised before being shared with the wider study team.

Analysis was by a group (n=8) of researchers and PPI (patient and public involvement) partners working together, using pooled data across sites. We took a reflexive thematic approach, generating themes in a broadly inductive way from the implicit and explicit ideas within participants’ accounts, following the six stages of analysis described by Braun and Clarke.^12^

### Patient and public involvement

Two public contributors (JG and RH-M) contributed to the TRIM research proposal and were members of the Research Management Group (RMG). JG chaired a TRIM patient panel of 10 members whose views on key study stages (eg, data collection, analysis, dissemination) were reported to the RMG for discussion. Support, in line with best practice, included honoraria, accessible information and a named individual (BAE) to facilitate public contributors’ effective involvement.^13 14^

## Results

### Participants

The role categories of the 25 participants are indicated in table 1, by site.

**Table 1:** Number of participants in the qualitative interviews, by site and role

	Site 1	Site 2	Site 3	Site 4
**Paramedic**	1	2	2	2
**Ambulance service manager**	2	2	1	1
**Call handler/dispatcher—EOC**	1	1	2	1
**Clinical advisor/manager—EOC**		1	1	2
**Emergency department clinician**	2		1	

EOC, Emergency Operations Centre.

## Themes

We present the findings structured around four themes, discussed below, illustrated with quotations coded by site and participant number. All themes were relevant to all sites, although the perspective of individual participants varied as indicated.

### Targeting the response

Changes made in triage processes in the EOC, including the adjustment of thresholds for response categories in line with demand, aimed to target ambulance service response to those people who were considered to benefit most, and to reduce infection risks associated with avoidable conveyance to hospital. The change in practice, particularly at the beginning of the pandemic, was major, with the highest of the levels of triage protocol described as ‘harsh’ by 2-03 Call handler/dispatcher–EOC, one of the people tasked with conveying perhaps unwelcome messages to callers seeking help.

Overall, although there was an increase in calls, the majority were resolved through telephone triage and advice and patients were not conveyed to hospital. For managers, this meant that demand pressures on crew and vehicle resources were eased:

The only people we were taking to hospital were people who needed to be taken … we were having many, many more phone calls … but because of Card 36, nearly none of that was coming to the crews. 1-06 Ambulance service manager

However, managers were also dealing with reduced supply of crews and vehicles, as job cycle times were extended through enhanced cleaning, and donning and doffing of personal protective equipment. In addition, a proportion of staff were off sick or isolating, so services aimed to protect crews from risk of infection wherever possible. One senior manager described how Card 36, in conjunction with additional locally developed ‘trigger questions’, supported this:

If there was strong suggestion through the various triage processes … that they were likely to be suffering from the pandemic disease, then you are less likely to send the face-to-face resource unless absolutely necessary. 2-05 Ambulance service manager

Participants described an expanded role for clinical advisors working in the EOCs, with additional staff taking on that role, with the aim of providing immediate advice and, wherever possible, closing the call without dispatch. However, presenting symptoms sometimes triggered an ambulance to be sent urgently, before there was a chance for the clinical advisor to have input in the EOC—a situation with potentially negative consequences:

They had to try and despatch a conveying resource to that patient within a particular timeframe, and that missed the opportunity to speak to a lot of these patients over the phone before sending a resource. And I’m not convinced that was the best, most appropriate response. Undoubtedly ambulance crews will have gone to Covid patients, picked up Covid and transmitted it to other patients. 1-05 Clinical Advisor–EOC

For paramedics and Emergency Medical Technicians (EMTs) attending patients, particularly challenging decisions needed to be made in relation to patients with COVID who were considered unlikely to survive. One paramedic described locally issued guidance about managing response which addressed this issue directly:

There were descriptions of certain patients with certain conditions at certain stages of their life. It would be pointless and fruitless to take them to an A&E department to die when they’re already in a nursing home … [Some colleagues] weren’t aware of new changes to policy and they’ve transported patients who, quite honestly, should have left them where they were to die in peace, in their care home, rather than die in hospital. 2-02 Paramedic

### Challenges for ambulance services as part of the wider healthcare system

The workload of emergency ambulance services, including the changes in response during the pandemic, was heavily shaped by what was going on in other parts of the local healthcare system. Participants talked about the challenges, particularly early in the pandemic, caused by changes in access to other parts of the healthcare system, leaving, as they saw it, ambulance services as a default option. There was a perception that patients had less opportunity to have input from primary care providers, as well as being less inclined to contact them due to public messaging about limiting strain on the health service. Ambulance service participants expressed frustration about this:

We had patients just leaping around the system, with nobody opening their doors to them, apart from the poor paramedics out there who were having to deal with this mess, caused by, you know, closing down services. 1-01 Ambulance service manager

We’ve ended up taking patients to hospital who we were fairly sure could have been managed by their GP in the community … If they catch Covid, they will die and they don’t need to be here. They could have been managed by primary care. It’s utterly frustrating. 2-02 paramedic

Although ED clinicians suggested that conveyance choices by ambulance crews were generally appropriate, they identified that on occasion patients were brought to the ED when they did not need hospital care:

We understood why the paramedics were bringing those patients into the ED, you guys have to work off different guidelines to us, but from a clinical perspective these were very well patients and we were happy to discharge them. 3-06 ED clinician

While bottlenecks at the ED were not unique to the pandemic, the additional pressures associated with responding to COVID-19 seemed to be a cause of tension:

You know, from our side, I think we tried to handle it as well as we can, but when we came up against like the A&Es, and sometimes it felt like us and them. And that just sort of caused a clash, then causes stress, poor working attitudes. 4-01 Paramedic

### The burden of constantly changing guidelines

Changes in processes in response to the pandemic were not a one-off, meaning that the labour involved in delivering changes was significant. Guidance was repeatedly revised and adjusted, particularly in the early months. Managers described being required to assimilate formal directives from external bodies such as Public Health England, sometimes combined with learning from colleagues in other ambulance services through established networks. There was then the task of communicating updates to colleagues:

We put in long, long hours … Because often in a day you’d have three or four process changes, based upon prevailing information, so to be there as a senior leadership team to be able to push those changes out in a rapid timely manner, but also be there to answer questions, was very difficult … God forbid you took a day off. 4-05 Manager–EOC

Staff working in the EOC, in turn, had to keep on top of the updates—a significant cognitive labour in itself, adding to the work involved in delivering the service response:

Every day was a learning curve, so every day something would slightly change. 3-05 Call handler–EOC

Paramedics and EMTs also had to update themselves on the frequent changes in guidelines, in practical terms a challenging task for staff based on the road.

### Emotional load of responding to the pandemic

The emotional demands placed on staff provide an important backdrop for their work to enact COVID-19-related changes, and participants talked about these extensively. For some participants, the overwhelming sense was of unhappy organisations:

Staff morale was awful. 2-04 Paramedic

The emotional load was experienced throughout ambulance services, not just among staff in patient-facing roles, and took many forms. Some participants described anxiety about colleagues who had fallen ill with COVID-19, or even died. Participants described the impact of the workload pressure itself, in terms of the hours, and the relentless, repetitive nature of responding to COVID-related calls:

It’s battered us… And it just grinds you down, or ground me down because it just repeats, repeats, repeats. 4-01 paramedic

Participants described how this pressure could combine with the risk of infection to themselves and a fear of taking the infection home, particularly when working in close proximity to colleagues:

It was probably the toughest thing I’ve ever done. And I was coming home and standing in the corridor taking my uniform off and running in the shower and sobbing my heart out day after day after day. 3-04 EOC call handler

Participants in patient-facing roles described the impact of exposure to the distress of patients and families, heightened by the restrictions placed on family members accompanying their loved one to hospital:

There was quite a few people we took in and we knew ourselves they’re probably never going to see that family again, and that was quite heart-breaking. So although the numbers of jobs didn’t increase … the physical and emotional impact [increased] massively. 4-04 Paramedic

Participants described the distress they felt at exposing non-COVID patients to the risk of infection, and from being unable to deliver the quality of care they would like to, particularly when waiting with patients in an ambulance outside hospital until the ED was ready to receive them:

And it was quite difficult from a wellbeing point of view … questioning the morals and ethics of what we were doing. 2-06 Paramedic and hospital liaison lead

Doing the best they could in the face of pressure and uncertainty, ambulance services were faced with a rapidly evolving situation unlike any they had faced before, with challenges in responding to the peaks and troughs of demand. Participants described having more high acuity patients in the mix:

It’s the makeup of the patients is different. And there are a greater number of category one and two patients [the more serious categories] … and probably less lower acuity business-as-usual condition. 2-05 Ambulance service manager

There was a sense from participants across sites and roles that ambulance services did as well as they could, in terms of identifying and conveying patients who needed hospital care, and also in terms of resilience and rapid adaptability. Faced with their existing resources being stretched, services found ways to maintain and expand the workforce as best they could, including, for example, bringing medical students in to handle 999 calls. Particularly among participants in senior ambulance service roles, there was considerable pride:

I think that we rose to the challenge during COVID, and we did an absolutely astounding job. And every single clinician on the ground, you know, the call takers, the dispatchers, just showed how flexible and how adaptable, and how resilient we are as a service. 1-01 Ambulance service manager

However, there were still concerns expressed by some participants about the sheer weight of demand placed on services, even beyond the initial waves of the pandemic:

It was absolutely horrendous I cannot put it into words there are no words to describe how bad it was certainly March April May … There wasn’t enough ambulances, there wasn’t enough paramedics, there wasn’t enough control staff, there wasn’t enough of anything. 3-04 EOC call handler

Covid’s still here [July 2021] and we are not coping, we’re drowning … We’re putting into place demand mitigation measures that aren’t really working … There’s still an avalanche, there’s still too much stuff coming down the mountain. It doesn’t matter how many guys you can put at the bottom with shovels, we don’t have enough guys. 1-06 ambulance service manager

## Discussion

We found that services made changes to triage practice using the best level of understanding available at the time, and in a healthcare setting which always operates in high levels of uncertainty. The adjustments allowed for flexibility in response models in the face of peaks and troughs in demand, with a shift towards more calls being dealt with remotely. Changes by other healthcare providers had an impact on the flow of patients to and from ambulance services. There was substantial work for staff at all levels in making changes to practice and processes in response to the pandemic. In addition, staff were carrying a heavy emotional load of anxiety, fear and moral distress. Nevertheless, we identified some sense of pride and resilience among participants in terms of how services coped with the uncertainty and at times overwhelming pressures of demand.

Our study adds to an emerging international literature exploring the role of ambulance services during the pandemic, and the experience of those working in them. To our knowledge, this is the first which describes the qualitative experience of implementing changes to triage and processes in UK ambulance services in response to COVID-19. It brings new insights into the experience of exacting changes to triage processes, adding to those studies which have addressed the impact of changes to triage processes.^15^ Other studies have documented changes in practice in relation to remote clinical advice, including an increase in home working^16^ and increased use of video consultation.^17^

Our findings on the emotional impact on ambulance service staff of working through the pandemic provide qualitative insights into the distress documented by Barrett *et al*,^18^ whose large-scale survey of paramedics in the UK found that 84% were at very high levels of risk of psychological distress in the first phase. Barrett *et al* found that one of the factors associated with the highest rates of risk of distress was frequently accessing guidelines (hourly rather than monthly). A qualitative study by Rees *et al*^19^ in one UK ambulance service documented paramedics’ experience of having to make ‘tragic choices’—professional, personal and societal—as they worked through the pandemic. Wankhade^20^ explored the emotional labour undertaken by ambulance service staff working through the pandemic in one UK service. The emotional impact has also been documented internationally, including studies conducted in the USA, Ireland, Iran, Canada and Australia.^21^,^25^ Our observations reflect the issues of moral distress, moral injury (the disconnect between a person’s beliefs or values and what they are asked to do), anxiety and burnout, which have been widely documented as part of the response to the COVID-19 pandemic in other healthcare settings.^26^

Our study brings new insight into the labour and cognitive load entailed in rapidly and repeatedly bringing about changes in working practice. While our participants emphasised both individual and organisational resilience, our study illustrates the importance, in any future pandemic, of acknowledging and mitigating the workload involved in implementing changes as well as the emotionally demanding context in which the changes are made. A strength of our study is that, while previous work has focused primarily on paramedics or reflected just on a single service, it explored the experience of staff in a range of work roles within multiple ambulance services and related their personal experience to the organisational changes which took place at system level. It allowed us to triangulate experience across the range of roles and to combine analysis across four different sites. The study is potentially limited by the fact that it includes just four of the UK regional ambulance services, and so may not have covered the full range of experience in different settings.

## Conclusion

The pandemic permitted bold changes in triage practice for emergency ambulance services, but also revealed heightened versions of familiar challenges: balancing protocols and judgement; dealing with unpredictable demand; and getting the interactions with other healthcare providers right. COVID-19 also presented staff with very particular challenges: the emotional impact on staff of doing their job through the pandemic; and the uncertainty and constant change in working practice which staff faced. Our study provides new insight to add to the still emerging literature on how emergency ambulance services responded to the challenges of COVID-19, which may inform organisational responses to any future pandemic.

## Data Availability

No data are available.
